# Who should support grieving children in school? Applying Winnicott's viewpoint to conceptualize the dyadic roles of teachers and school mental-health professionals in the context of pediatric grief

**DOI:** 10.3389/fpsyt.2023.1290967

**Published:** 2023-11-06

**Authors:** Rivi Frei-Landau

**Affiliations:** The Loss, Crisis, and Resilience in a Multicultural Lens Research Lab, Faculty of Psychology, Achva Academic College, Shikmim, Israel

**Keywords:** Winnicott, mental health, loss, pediatric grief, school-based support

## Introduction

Coping with grief after loss has been at the forefront of concerns amidst the COVID-19 pandemic (1–3). In a recent study (4), it was estimated that due to the pandemic more than 1.5 million children globally lost a caregiver. Childhood bereavement may profoundly impact child development (5). It is associated with heightened risks of impaired academic and social performance (6), mental health issues (7, 8), substance use disorders (9), and higher mortality rates (10). Yet children are at risk of receiving no post-loss support (11–13), as in many countries there are no structured, official guidelines regarding different caregivers' roles. Scholars have thus advocated the need to define and clarify the roles of teachers and other school staff, such as SMPs (14).

This paper draws upon the well-established concepts of Winnicott, who significantly contributed to the field of child development. Applying Winnicott's perspective on the role of the caregiver dyad (i.e., parents), I would suggest an analogy between the father-mother role (when supporting the evolving infant's development) and the dyadic SMP-teacher role, to conceptualize what I believe should be the role of each of these two entities when working together to support grieving children's development.

## Childhood bereavement and caregivers' roles

According to childhood bereavement estimation models in the US, by age 18, one in 14 children (7.2%) will experience the death of a parent or sibling (15), and 90% will experience the death of a close friend or relative (5). A review (16) indicated that bereaved children are particularly vulnerable and that adequacy of care from those around the child is essential to their healthy development.

### SMPs' role in the context of childhood bereavement

Scholars (5) argue that school mental-health professionals (SMPs) are well-suited to identify and support grieving students; to provide guidance to peers; and to offer teachers' training. Yet instances of loss and trauma are constantly occurring worldwide, resulting in SMPs facing continuous stress and emotional overload (17), potentially preventing them from being available to bereaved children and turning teachers into the main school caregivers.

### Teacher's role in the context of childhood bereavement

Seventy percent of American teachers reported having at least one grieving student in their classroom (18). Although school-based support has been found to facilitate grieving students' adjustment both in the US (5) and Europe (19, 20), school support is not coordinated (21). In fact, the question regarding teachers' role is controversial. On the one hand, the school is argued to be a “secure secondary family,” and that educators are well-placed to monitor mental health, and support academic and social issues (22–25). On the other hand, studies showed (14) that although most teachers recognized the need to acknowledge children's grief, they claimed that they were not trained to do so, and were overloaded with pedagogical tasks. Hence, a “crisis plan,” for which the entire school personnel team is trained and prepared to implement, is needed (26). Yet, to build such a plan, conceptualizations must be formulated about the different roles of different school personnel in order to best integrate these role expectations into practice.

## Winnicott's viewpoint on caregivers' roles

D.W. Winnicott, a British pediatrician, made significant contributions to the field of child development. A well-known metaphor developed by Winnicott is the “holding environment”—the emotional and physical space provided by the caregiver, typically the mother, protecting the baby by holding him/her in her arms. The caregiver provides an environment that literally and figuratively holds the child, enabling the infant to feel safe. A holding space therefore means being fully present, as one sits with another through a difficult time.

In terms of who provides a holding environment for whom, scholars (27, 28) have argued that, according to Winnicott (29), the father's role with the newborn is protecting the mother-child relationship. He provides a secure environment for the mother, enabling her to provide a nurturing environment for the baby. The father can offer both physical (practical) support, and emotional support, allowing the mother to cope better with motherhood's frustrations. In some cases, the father must provide parenting for the mother, if she is struggling with the demands of motherhood (30). Fathers can also serve as a “mother substitute,” providing the baby with sensitive and responsive care, if the mother needs a respite. Eventually, a strong relationship between the father and mother provides the infant with security—a “rock to which he can cling” (29).

I thus wish to argue that, in the school setting, the role of the SMP should be similar to that of the father, in accordance with Winnicott's claim that “as the mother feels supported, she can better respond to the infant's needs.” Namely, the SMP should support the teacher so that the teacher can support the grieving student.

## The voices of grieving students and their teachers

In my prior extensive research on issues of grief and childhood bereavement, and particularly among teachers^1^ (23, 24, 31–33), I have interviewed both teachers and students and noted that although SMPs are expected to provide support, the students themselves often don't want it. In fact, they tend to prefer to be supported by their teachers since they have prior relationship with them. Furthermore, considering the social stigma around requesting help from SMPs, students are sometimes reluctant to approach them at school, as opposed to teachers, with whom a conversation is perceived as “normal.” With regard to teachers, they often perceive their roles as child-supporters, yet they often say they need support themselves in order to continuously support their students. In a recent study teachers reported of three support needs when managing pediatric grief: knowledge—both theoretical and practical, acknowledgment of their own emotional coping, and support from mental health professionals in school (23). Other studies emphasized the need for clear policy and the importance of school community when managing pediatric grief in school (21, 34).

## Discussion: who should support grieving children at school?

Grieving children have various support needs from school, such as support in academic and social challenges aroused by the grief that require long-term support (34). Similarly, teachers report of having various needs when supporting their grieving students (23). So, looking at the broader picture, what is the role of SMPs and teachers in the context of pediatric grief in school? Who and how should support grieving children in school?

It seems that the most reasonable and effective way to help the grieving child is to provide a holding environment similar to that suggested by Winnicott: The SMPs should support the teachers by offering both practical and emotional support, so that the teachers can better support the grieving child. Nevertheless, as teachers are not trained to be therapists, this arrangement should only be applied in the context of “typical” grief reactions. If a child presents a prolonged grief (i.e., complicated grief), and/or needs extensive professional therapy, then the SMP would be the primary caretaker and the teacher the secondary one. This notion accords with Winnicott's claim that the father can also be a “primary caregiver” when needed.

**Figure 1:**
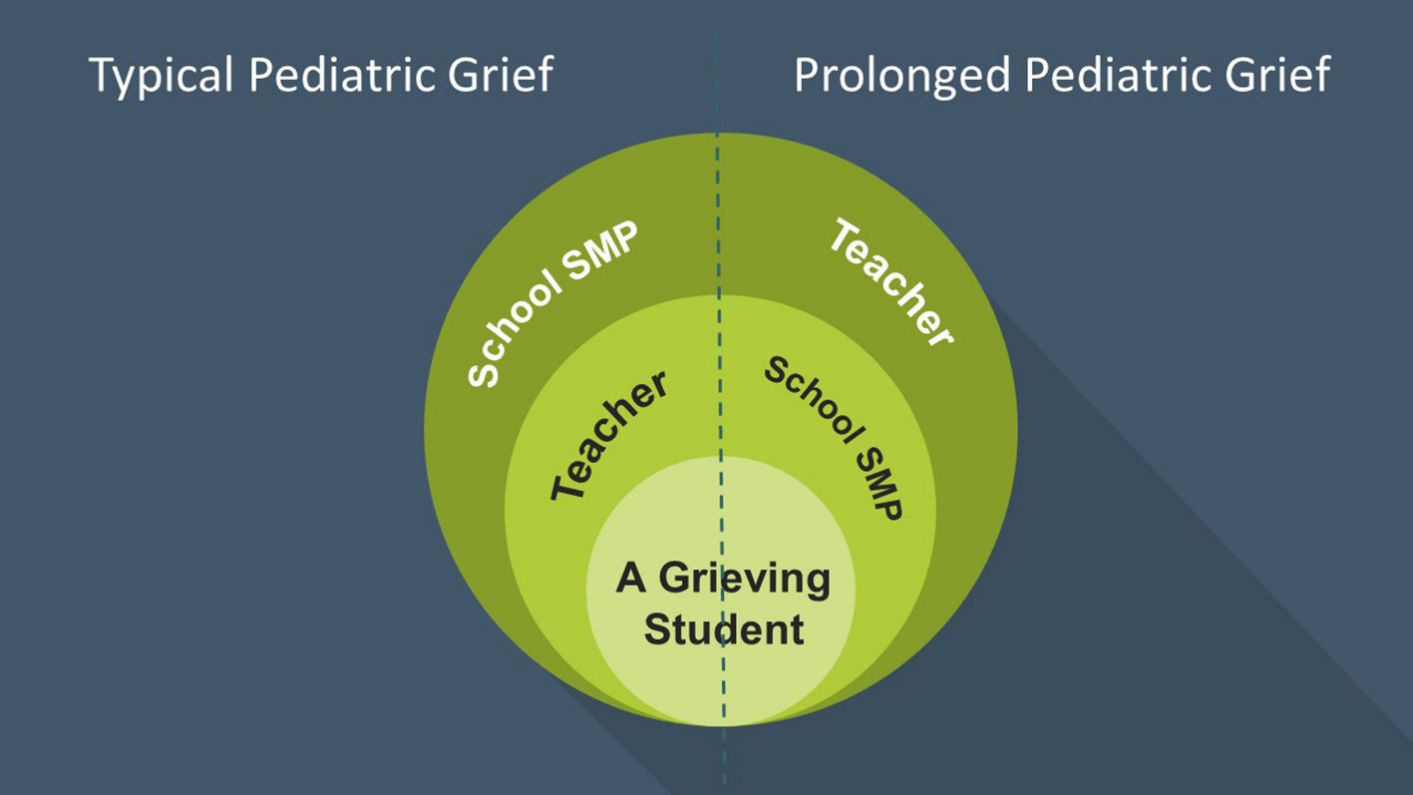
Conceptualized roles of SMPs and teachers when supporting grieving children.

Figure 1 illustrates the roles of SMPs and teachers in pediatric grief situations. In a “normal” grief situation the teacher envelops the grieving student while being enveloped by the SMP. In a “prolonged” grief situation, the situation is reversed.

This suggested model aligns with the claim that children are more likely to open up to a teacher they know and trust, as well as with studies showing the importance of school leadership supporting school personnel (35). Yet this model's innovation lies in its unique conceptualization drawn upon Winnicott's viewpoint, enabling a better use of resources when managing pediatric grief in schools.

## Implications

This conceptualization has several implications. First, teachers should receive training focused on the *child*'s understanding of death and the child's grief-needs, whereas SMPs should receive training focused not only on the *child*'s understanding/needs but also on the *teachers'* needs in coping with supporting these students. The difference is analogous to that between being trained to become a therapist vs. being trained to become a supervisor. Another implication involves policy. Specifically, in most cases, SMPs should provide support to teachers, and teachers should provide support to students. This leads to a more economic management of grief in school and will also enable SMPs to be truly available in cases of “complicated grief.”

In conclusion, the conceptualized model may substantially contribute to the effective support of bereaved children at school. It may also promote policy changes that clarify caregivers' unique roles and practices, to optimally support grieving children's development.

## Author contributions

RF-L: Conceptualization, Visualization, Writing—original draft, Writing—review & editing.

## Footnotes

^1^Prior research was conducted after receiving Achva Academic College's ethic committee's approval (no. 153)

## References

[1] Frei-Landau R. When the going gets tough, the tough get - creative: Israeli Jewish religious leaders find religiously innovative ways to preserve community members' sense of belonging and resilience during the COVID-19 pandemic. Psychol Trauma. (2020) 12:S568. 10.1037/tra0000822 32538652

[2] Kozato N Mishra M Firdosi M. New-onset psychosis due to COVID-19. BMJ Case Rep. (2021) 14:e242538. 10.1136/bcr-2021-242538 PMC8076917 33906867

[3] Moideen S Thomas R Kumar PS Uvais NA Katshu MZ. Psychosis in a patient with anti-NMDA-receptor antibodies experiencing significant stress related to COVID-19. Brain, Behav Immun Health. (2020) 7:100125. 10.1016/j.bbih.2020.100125 32835301 PMC7415166

[4] Hillis SD Unwin HJ Chen Y Cluver L Sherr L Goldman PS. Global minimum estimates of children affected by COVID-19-associated orphanhood and deaths of caregivers: a modelling study. Lancet. (2021) 398:391–402. 10.1016/S0140-6736(21)01253-8 34298000 PMC8293949

[5] Schonfeld DJ Demaria TP. The role of school psychologists in the support of grieving children. School Psychol Quart. (2018) 33:361–2. 10.1037/spq0000286 30234358

[6] Brent DA Melhem NM Masten AS Porta G Payne MW. Longitudinal effects of parental bereavement on adolescent developmental competence. J Clin Child Adoles Psychol. (2012) 41:778–91. 10.1080/15374416.2012.717871 23009724 PMC3493857

[7] Kaplow JB Saunders J Angold A Costello EJ. Psychiatric symptoms in bereaved versus nonbereaved youth and young adults: a longitudinal epidemiological study. J Am Acad Child Adolesc Psychiatry. (2010) 49:1145–54. 10.1097/00004583-201011000-00008 20970702 PMC2965565

[8] Margoob MA Rather YH Khan AY Singh GP Malik YA Firdosi MM. Psychiatric disorders among children living in orphanages—Experience from Kashmir. JK-Practitioner. (2006) 13:S53–S55.

[9] Otowa T York TP Gardner CO Kendler KS Hettema JM. The impact of childhood parental loss on risk for mood, anxiety and substance use disorders in a population-based sample of male twins. Psychiat Res. (2014) 220:404–409. 10.1016/j.psychres.2014.07.053 25146695 PMC4385714

[10] Li J Vestergaard M Cnattingius S Gissler M Bech BH Obel C. Mortality after parental death in childhood: A nationwide cohort study from three Nordic countries. PLoS Med. (2014) 11:e1001679. 10.1371/journal.pmed.1001679 25051501 PMC4106717

[11] Albuquerque S Santos AR. “In the same Storm, but not on the same Boat”: children grief during the COVID-19 pandemic. Front Psychiatry. (2021) 12:638866. 10.3389/fpsyt.2021.638866 33574777 PMC7870701

[12] Shalev R Dargan C Abdallah F. Issues in the treatment of children who have lost a family member to murder in the Arab community in Israel. OMEGA-J Death Dying. (2021) 83:198–211. 10.1177/0030222819846714 31039665

[13] Weinstock L Dunda D Harrington H Nelson H. It's complicated—adolescent grief in the time of COVID-19. Front Psychiat. (2021) 12:638940. 10.3389/fpsyt.2021.638940 PMC7940762 33708148

[14] Dyregrov A Dyregrov K Idsoe T. Teachers' perceptions of their role facing children in grief. Emot Behav Diffic. (2013) 18:125–134. 10.1080/13632752.2012.754165

[15] Burns M Griese B King S Talmi A. Childhood bereavement: understanding prevalence and related adversity in the United States. Am J Orthopsychiat. (2020) 90:391–405. 10.1037/ort0000442 31999137

[16] Stroebe M Schut H Stroebe W. Health outcomes of bereavement. Lancet. (2007) 370:1960–73. 10.1016/S0140-6736(07)61816-9 18068517

[17] Levkovich I Ricon T. Understanding compassion fatigue, optimism and emotional distress among Israeli school counsellors. Asia Pacific J Counsel. (2020) 11:159–80. 10.1080/21507686.2020.1799829

[18] American Federation of Teachers. Grief in the Classroom. New York Life Foundation (2012). Available online at: https://www.newyorklife.com/assets/foundation/docs/pdfs/NYL-AFT-Bereavement-Survey.pdf

[19] Dyregrov A Dyregrov K Lytje M. Loss in the family – A reflection on how schools can support their students. Bereav Care. (2020) 39:95–101. 10.1080/02682621.2020.1828722

[20] McLaughlin C Lytje M Holliday C. Consequences of childhood bereavement in the context of the British school system. Faculty of Education, University of Cambridge. (2019).

[21] Dimery E Templeton S. Death, bereavement and grief: the role of the teacher in supporting a child experiencing the death of a parent. Practice. (2021) 3:146–65. 10.1080/25783858.2021.1882263

[22] Frei-Landau R. Lost When Facing Loss: Why Educate Teachers about Loss Death? [Video]. TEDx (2021). Available online at: https://www.ted.com/talks/dr_rivi_frei_landau_lost_when_facing_loss_why_educate_teachers_about_loss_and_death

[23] Frei-Landau R Mirsky C Sabar-Ben-Yehoshua N. Teachers' coping with grieving children during the COVID-19 pandemic. In:Peleg O Shalev R Hadar E, editors. Educational Counseling in Stressful Life Events, Crisis and Emergency. Pardes Publishing (2023).

[24] Frei-Landau R Abu-Much A Sabar-Ben Yehoshua N. Religious meaning making of Muslim parents bereaved by homicide: Struggling to accept 'God's will' and longing for ‘Qayama' day. Heliyon. (2023) 9:e20246. 10.1016/j.heliyon.2023.e20246 37809798 PMC10560023

[25] Holland J Wilkinson S. A comparative study of the child bereavement response and needs of schools in North Suffolk and Hull, Yorkshire. Bereavement Care. (2015) 34:52–8. 10.1080/02682621.2015.1063858

[26] Lytje M. The success of a planned bereavement response–a survey on teacher use of bereavement response plans when supporting grieving children in Danish schools. Pastoral Care Educ. (2017) 35:28–38. 10.1080/02643944.2016.1256420

[27] Faimberg H. The paternal function in Winnicott: The psychoanalytical frame. Int J Psychoanaly. (2014) 95:629–40. 10.1111/1745-8315.12236 25229543

[28] Reeves C. On the margins: The role of the father in Winnicott's writings. In: Donald Winnicott today. Routledge (2012). p. 358–85.

[29] Winnicott DW. The theory of the parent-infant relationship. In:Winnicott DW, editor. The maturational processes of the facilitating environment: Studies in the theory of emotional development. International Universities Press (1960). p. 37–55. 10.4324/9780429482410-3

[30] Tuttman S. The father's role in the child's development in the capacity to deal with separation and loss. J Am Acad Psychoanaly. (1986) 14:309–22. 10.1521/jaap.1.1986.14.3.309 3744953

[31] Frei-Landau R Tuval-Mashiach R Silberg T Hasson-Ohayon I. Attachment to god among bereaved Jewish parents: Exploring differences by denominational affiliation. Rev Relig Res. (2020) 62:485–96.

[32] Frei-Landau R Hasson-Ohayon I Tuval-Mashiach R. The experience of divine struggle following child loss: the case of Israeli bereaved Modern-Orthodox parents. Death Stud. (2022) 46:1329–43. 33259263 10.1080/07481187.2020.1850547

[33] Levitan F Frei-Landau R Sabar-Ben-Yehoshua NA. “I Just Needed a Hug”: culturally-based disenfranchised grief of Jewish ultraorthodox women following pregnancy loss. OMEGA-J Death Dying. (2022) 10.1177/00302228221133864 36227857

[34] Lytje M. Voices we forget—Danish students experience of returning to school following parental bereavement. OMEGA-J Death Dying. (2018) 78:24–42. 10.1177/0030222816679660 30286685

[35] Demaria T Gilman R Mazyck D Schonfeld D. The impact of distress, personal meaning and training on the delivery of support to grieving students by school nurses. J Sch Nurs. (2021) 7:10598405211041299. 10.1177/10598405211041299 34490823

